# Triple-Networked Hybrid Hydrogels Reinforced with Montmorillonite Clay and Graphene Nanoplatelets for Soft and Hard Tissue Regeneration

**DOI:** 10.3390/ijms232214158

**Published:** 2022-11-16

**Authors:** Anuj Kumar, So-Yeon Won, Ankur Sood, So-Yeon Choi, Ritu Singhmar, Rakesh Bhaskar, Vineet Kumar, Sun Mi Zo, Sung-Soo Han

**Affiliations:** 1 School of Chemical Engineering, Yeungnam University, Gyeongsan 38541, Republic of Korea; 2 Research Institute of Cell Culture, Yeungnam University, Gyeongsan 38541, Republic of Korea; 3 School of Mechanical Engineering, Yeungnam University, Gyeongsan 38541, Republic of Korea

**Keywords:** hydrogels, triple network, montmorillonite, graphene nanoplatelets, tissue engineering

## Abstract

Hydrogel is a three-dimensional (3D) soft and highly hydrophilic, polymeric network that can swell in water and imbibe a high amount of water or biological fluids. Hydrogels have been used widely in various biomedical applications. Hydrogel may provide a fluidic tissue-like 3D microenvironment by maintaining the original network for tissue engineering. However, their low mechanical performances limit their broad applicability in various functional tissues. This property causes substantial challenges in designing and preparing strong hydrogel networks. Therefore, we report the triple-networked hybrid hydrogel network with enhanced mechanical properties by incorporating dual-crosslinking and nanofillers (e.g., montmorillonite (MMT), graphene nanoplatelets (GNPs)). In this study, we prepared hybrid hydrogels composed of polyacrylamide, poly (vinyl alcohol), sodium alginate, MMT, and MMT/GNPs through dynamic crosslinking. The freeze-dried hybrid hydrogels showed good 3D porous architecture. The results exhibited a magnificent porous structure, interconnected pore-network surface morphology, enhanced mechanical properties, and cellular activity of hybrid hydrogels.

## 1. Introduction

Tissue engineering is the most preferred strategy to repair damaged biological tissues by growing cells on scaffolds using bioactive molecules (e.g., growth factors) [1]. In this regard, tissue-repairing scaffolds have been extensively manufactured in anticipation of obtaining a perfect scaffold structure for mimicking the typical features of a native tissue 3D environment (soft or hard tissue) [2,3]. The biomaterial scaffold offers a favorable 3D environment, carries cells, is stable, and delivers pro-tissue regeneration factors. On the other hand, the scaffold presents certain restrictions in terms of timely degradation, toxicity, and immunomodulatory concerns. Therefore, the design and manufacturing of the biomaterial scaffolds is one of the crucial factors in tissue engineering applications. Regeneration of tissue is a complex phenomenon and prerequisite to understand the biological architecture while designing tissue-engineered biomaterial scaffolds for satisfactory results [4]. To manufacture scaffolds, the decisive features such as the suitability of their mechanical performance in the implantation site, satisfactory triggering of the immune reaction, and the nature and spatio-temporal allocation of the scaffold’s properties must be taken into consideration. The precise manipulation of these features in the scaffold is the influential approach to regulate cell fate, directing cellular performance and the response in vitro and in vivo (e.g., cell attachment, viability and proliferation, differentiation, matrix production) [5]. In this regards, manipulation of these features in the scaffold can be categorized as (1) alteration of the material properties (e.g., chemistry, morphology/geometry, pore construction, and stiffness); (2) the design of the scaffold composition for appropriate interaction with bioactive molecules; (3) the variation in designed scaffolds for the effective supply of signaling molecules; and (4) the manufacturing of the scaffold for the administration of bioactive molecules and cellular activity [6].

Compared to stable scaffold structures such as films, patches, etc., hydrogels with their dynamic behavior are a potent candidate as they provide a moist 3D environment for cell attachment and growth along with support in the transportation of nutrients and elimination of waste materials across hydrogels, thereby imitating the 3D microenvironment. In brief, hydrogels are highly hydrophilic 3D networks and can withhold a substantial amount of water or biological fluids by maintaining their original construct [7]. Hydrogels are very promising functional biomaterials’ scaffolds due to their chemical and mechanical attributes, most similar to native tissues [8,9]. However, their soft nature, low mechanical properties, and unsatisfactory degradation profile limits their intense exploitation in various tissue engineering applications, especially for load-bearing tissue regeneration. Therefore, the development of novel, stronger, and more durable hydrogels with enhanced biocompatibility and multifunctionality is highly desired [10]. A pristine biomaterial (natural or synthetic) does not provide sufficient properties to develop native-like 3D tissue microenvironment (i.e., hydrogel), because biopolymers and synthetic polymers exhibit several limitations apart from the useful characteristics such as good water solubility, excellent biocompatibility, biodegradability, and low immunogenicity. For example, animal-based biopolymers from animals exhibit low reproducibility, high production cost, and low mechanical properties. On the other hand, synthetic polymers possess high reproducibility, low cost, outstanding mechanical properties, ease of functional modification; however, they have limited water solubility, risk of an inflammatory response, unsatisfactory cytocompatibility, and the lack of cell recognition sites [10]. Among natural polymers, polysaccharides are believed to have more promising functional characteristics, such as their renewability, low cost, and good biocompatibility [11,12] and remarkably could be studied in developing composite/nanocomposite hydrogels with satisfactory features for tissue regeneration. Apart from other promising features such as dynamicity, the mechanical attributes of the hydrogel networks are a much needed component for tissue regeneration. The mechanical performance of hydrogels could be improved in different potential approaches, wherein the use of nanofillers is effective in providing better or synergistic characteristics for satisfactory tissue regeneration as compared to pristine biomaterials. In the past few decades, hydrogels composed of natural or synthetic polymers or their combination have broadly been developed for diverse tissue engineering applications.

Polyacrylamide (PAM) has also been utilized in preparing tissue engineering hydrogels owing to its hydrogel forming ability and suitable biocompatibility [13], but pristine PAM-based hydrogels are mechanically soft and brittle, which impedes their use in tissue engineering. Furthermore, among different polymer categories, poly (vinyl alcohol) (PVA) and alginate (AG) together have shown huge prospects for fabricating biomaterial composites for biomedical applications [14]. PVA, with remarkable physical properties and chemical resistance/inertness, has been applied in the tissue engineering area due to its cheaper cost, significant hydrophilicity, reasonable mechanical properties, satisfactory cytocompatibility, and hydrogel forming ability [13]. In addition, AG is also hydrophilic and presents important characteristics such as excellent biocompatibility, biodegradability, and biostability that are appropriate for biomedical applications [15]. AG shows a broad diversity in chemical compositions and presents various gelling properties [16,17]. This intrinsic viscoelasticity of AG differs in agreement with the originated building blocks (M/G ratio), wherein a high amount of G blocks in AG creates brittle and strong gels, while a higher amount of M block in AG causes shrinkage in the gel network and superior flexibility. Moreover, the gel formation of AG is difficult if the molar fraction of G block is less than 20–25% [18,19]. AGs exhibit an aspect of gel formation (i.e., ionic gelation) in the presence of mono valent (e.g., H^+^), divalent (e.g., Ca^2+^, Fe^2+^, Zn^2+^), and trivalent (e.g., Fe^3+^, Al^3+^) cations through different reaction mechanisms. Among them, Ca^2+^-induced gelation is the most common approach and selectively binds to the G blocks (i.e., the egg-box model). Among various AGs, sodium alginate (SAG) has been widely explored and analyzed for pharmaceutical and biomedical applications [14]. Among the inorganic nanostructures, the incorporation of nanoclay or clay minerals presents low cost and good biocompatibility, for example, montmorillonite (MMT) nanoclay [20]. MMT has been used as the most efficient hemostatic vehicle [21]. MMT is layered with hydrated aluminum silicate (Mx (Al_4_-xMg_x_) Si_8_O_20_(OH)_4_; known as smectite) constituted with one O-Al (Mg)-O octahedral sheet placed between two O-Si-O tetrahedral sheets [22]. MMT has shown therapeutic behavior [23] as well as accelerated tissue regeneration ability, stimulation of blood coagulation, and also exhibited antibacterial activities, because it is adsorbed on bacteria (Escherichia coli and Staphylococcus aureus) and neutralizes their toxins [24,25]. MMT has been shown to be nontoxic, biocompatible, and internalized by cells through endocytosis making it a potential candidate in biomedical applications [26,27]. In addition, graphene and its different forms (e.g., graphene nanoribbons (GNRs) or graphene nanoplatelets (GNPs) or graphene nano-onions (GNOs)) have received great attention as nanofillers to improve the electrical conductivity and mechanical properties. In addition, they are non-toxic at low fractions (below or up to the concentrations of 50 µg/mL) [28] and can also be influenced by the agglomeration of particles. Moreover, GNPs show good biocompatibility, high surface area, and remarkable electrical properties [29]. GNPs can impart high mechanical strength to the scaffold owing to its multilayer arrangement though; the ability to provide mechanical reinforcement to the scaffold depends on various attributes of the graphene nanofillers which includes the lateral size, thickness, and oxidation degree [30]. It has also been reported that the addition of GNPs increases the biodegradability of scaffolds due to an increment in porous behavior, which in turn increases the contact surface of the scaffold with the buffered solution. In addition, the ability of GNPs to act as compatibilizer reduces the size and increases the numbers, causing the degradation [31]. However, the use of GNPs is limited due to their hydrophobicity and high agglomeration tendency, but the re-stacking of nanosheets could be prevented by incorporation into polymeric or ceramic systems [32,33]. Herein, we consider the above-mentioned biomaterials to fabricate composite hydrogels with adjustable physical and mechanical characteristics as well as the biological potential for tissue regeneration. The synergistic effect of gelling characteristics of SAG and crystalizing quality of PVA is very influential in improving the physicochemical and biological properties of hydrogel scaffolds [34,35]. Therefore, a PAM hydrogel network was modified with a PVA/SAG polymeric system followed by further addition of MMT and/or GNPs to improve the tissue-like elasticity, bioactivity, and mechanical properties of the composite hydrogels. In this case, PVA and SAG assist in creating immense volume of physical crosslinking sites (i.e., crystallites) and ionic gelation among molecular chains of the PAM hydrogel network to facilitate stiffness, toughness, and elastic behaviors.

In the present study, PAM/PVA-SAG dynamic hydrogel networks were developed through free-radical polymerization via borax crosslinking (B(OH)_4_^−^) and/or ionic crosslinking (i.e., Ca^2+^ ions), which were further incorporated with MMT or MMT/GNPs as nanofillers to enhance the physical, mechanical, and biological properties of the composite hydrogels. Here, triple-networked hydrogel is formed through covalently crosslinked PAM as the first network and PVA matrix as the second network formed via chain crystallization and borax crosslinking in connection to the first network. Further, SAG-Ca^2+^ could be defined as the third network formed through physical and ionic crosslinking. Herein, the synergistic effects of MMT and GNPs on physicochemical, mechanical, and biological properties (in vitro) of hybrid hydrogels was investigated. Hybrid hydrogels were characterized by scanning electron microscopy (SEM), Fourier-transform infrared (FTIR) spectroscopy, X-ray diffraction (XRD), swelling and degradation studies, mechanical properties (under compression), and cell culture studies with pre-osteoblast and fibroblast cells (morphology using SEM and live/dead assay, and quantification using MTT assay). This study exhibited promising outcomes for the possible application of the developed hybrid hydrogels in soft and hard tissue regeneration.

## 2. Results and Discussion

### 2.1. Formation of Hybrid Hydrogels

**Figure 1:**
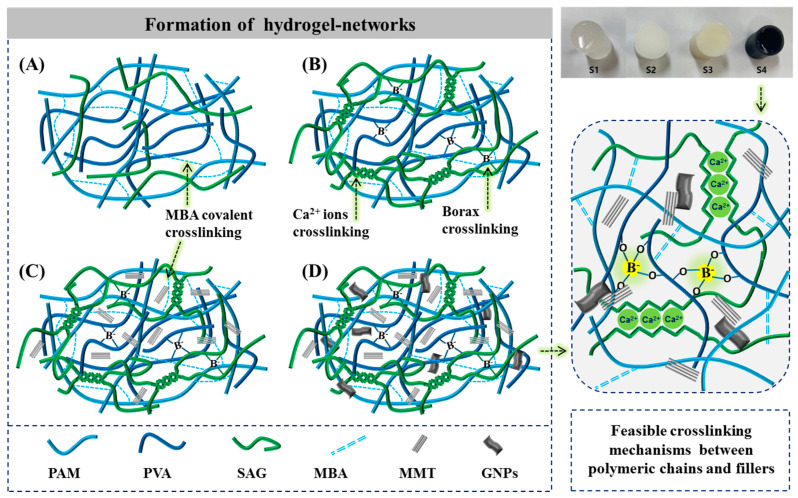
Schematic illustration of the formation of hydrogel networks: (**A**) PAM-g-(PVA/SAG); (**B**) PAM-g-(PVA/SAG)/BX/Ca^2+^; (**C**) PAM-g-(PVA/SAG)/BX/Ca^2+^/MMT; and (**D**) PAM-g-(PVA/SAG)/BX/Ca^2+^/MMT-GNPs) designated as S1, S2, S3, and S4, respectively. (* Hydrogen bonding is not shown here).

Figure 1 shows the schematic of the crosslinking mechanisms within the hydrogel networks. Figure 1A presents the covalently polymerized hydrogel network of PAM with interpenetrated network of PVA and SAG chains (S1), whereas Figure 1B demonstrates the hybrid hydrogel network with borax (B(OH)_4_^−^) crosslinking and ionic (Ca^2+^) interactions through the egg-box model (S2). The uniform distribution of MMT is shown within the crosslinked hybrid hydrogel network (S3) in Figure 1C, and the dispersion and interaction of both MMT and GNPs is shown within the hybrid hydrogel network (S4, Figure 1D). In the case of the S1 hydrogel (A), the polyacrylamide-based hydrogel network containing an interpenetrating network of PVA and SAG chains was developed via covalent crosslinking and hydrogen bonds. Furthermore, in the S2 hydrogel network (B), the crosslinking with both B(OH)_4_^−^ (with adjacent -OH groups) and Ca^2+^ ions (with -COOH groups) was introduced, wherein (1) B(OH)_4_^−^ (aq) ions favored to create a complex system (reversible and exchangeable ionic interaction in the solution state [36]) because B-O boundaries are between full ionic and full covalent bonding (weak and labile), whereas Ca^2+^ ions provide uncontrolled rapid crosslinking (inhomogeneous ionic interactions) with SAG molecular chains. The synergistic crosslinking effect facilitated the formation of a viscoelastic and rigid hybrid hydrogel network, respectively. Additionally, the loading of MMT clay (S3) or the binary system of MMT/GNPs (S4) further enhanced the physical crosslinking site within the hydrogel network. Moreover, the combination of crosslinking agents and nanofillers improved the overall stability of the composite hydrogels.

### 2.2. Structural Analysis

**Figure 2:**
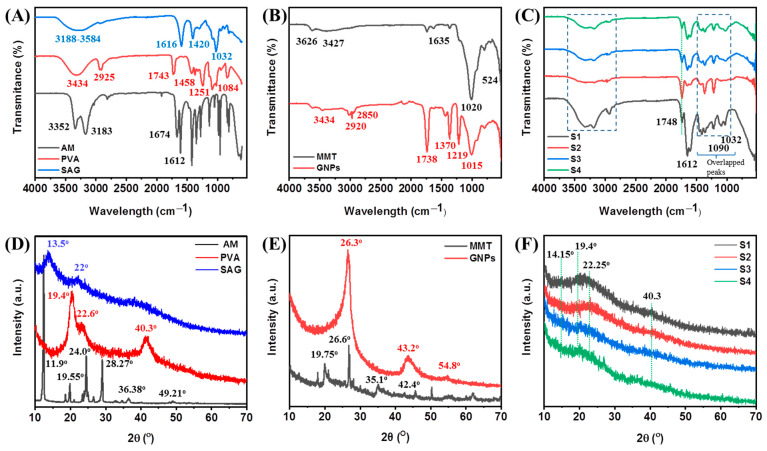
Structural analysis of the components and hybrid hydrogels: FTIR spectra (**A**–**C**) and XRD patterns (**D**–**F**) of the components (AM, PVA, SAG, MMT, and GNPs) and hydrogels (S1, S2, S3, and S4), respectively.

FTIR was employed to evaluate the molecular structures of pristine components and their intermolecular interactions in hybrid hydrogels. Figure 2A,B shows the typical characteristic peaks of monomers/polymers (AM, PVA, and SAG) and nanofillers (MMT and GNPs). In Figure 2A, the characteristic peaks for AM are observed at 3352 cm^−1^ and 3183 cm^−1^ (-NH_2_ stretching), 1674 cm^−1^, and 1612 cm^−1^ (C=O and C=C stretching), which after polymerization (in PAM) can be seen at around 3341 cm^−1^ and 3201 cm^−1^ (-NH_2_ stretching), 1666 cm^−1^ (C=O stretching, amide-I), 1608 cm^−1^ (-NH_2_ in-plane deformation in -CONH_2_, amide-II), 1409 cm^−1^ (C-N stretching), and 1125 cm^−1^ (-NH_2_ in-plane rocking). For PVA, peaks are observed at around 1084 cm^−1^ (strong) which could be assigned to the crystalline domain of PVA (C-O stretching), further peaks at 1458 cm^−1^ (-CH_2_ bending), 1743 cm^−1^ (C=O stretching from non-hydrolyzed ester groups), and 3434 cm^−1^ (-OH groups) were also recorded [13]. Further, major IR peaks of SAG are observed at around 3188–3584 cm^−1^ (-OH groups), 1650 cm^−1^, and 1616 cm^−1^ (due to C=O stretching from carboxylate (-COO^−^) groups) and 1420 cm^−1^ (symmetric stretching of carboxyl anions salt groups), and 1032 cm^−1^ (C-O-C stretching from cyclic ether bridge) [36]. For both nanomaterials, major IR peaks of MMT are observed at around 3626 cm^−1^ (Al-O-H stretching), 1635 cm^−1^ (Al-O-H bending), 1093–1020 cm^−1^ (Si-O-Si stretching), 915–500 cm^−1^ (due to oxide bands of metals, such as Al, Si, and Mg), respectively [37]. The major peaks of GNPs are observed at 3434 cm^−1^ (assigned to the OH stretching from the carboxylic and phenolic groups), while two other IR peaks at 2920 cm^−1^ and 2850 cm^−1^ are assigned to the aliphatic compounds that originated on the GNP surface, respectively. In addition, the characteristic peak due to C=O stretching (from ketone, lactone, and carboxyl groups) was observed at 1738 cm^−1^ and 1680 cm^−1^, respectively. Further, the peak at 1370 cm^−1^ is assigned to the C-OH stretching vibration [38]. The effect of crosslinking the hybrid hydrogel S1 separately with borax and Ca^2+^ on the structural attributes is shown in Figure S1 (Supplementary Materials).

In the case of the hybrid hydrogel of PAM, PVA, and SAG, these characteristic peaks are overlapped and exhibited significant changes in the fingerprint region (1500–1000 cm^−1^) after polymerization (Figure 2C; S1) and crosslinking reaction (Figure 2C; S2). In the crosslinked hybrid hydrogel (S2), a shifting in the main characteristic peaks could be observed due to the formation of new functionality. Some peaks are shifted to higher wavenumbers due to the formation of Ca^2+^ complex (egg-shaped system with bidentate bridging or chelating) and non-covalent bonding between borax and -OH groups (nature of bonding is not absolutely clear) of the components, while some characteristic peaks are shifted to lower wavenumber due to intra-/inter-molecular hydrogen bonds. The interaction of Ca^2+^ ions with SAG showed the reduction in peak intensity and the changes in positions, particularly the peak from 1420 cm^−1^ (symmetric stretching of carboxyl anions salt groups, -COO^−^Na^+^) to 1442 cm^−1^ (-COO^−^(Ca^2+^)-OOC^−^) [36]. In addition to these changes, the presence of borax (B(OH)_4_^−^) and borate complex can be seen at around 1425 and 1335 cm^−1^ (asymmetric relaxation of B-O-C linkages), 878 cm^−1^ (B-O stretching from residual B(OH)_4_^−^), and 652 cm^−1^ (bending of B-O-B linkages) [34]. Furthermore, after the incorporation of MMT (Figure 2C; S3) or MMT/GNPs (Figure 2C; S4), these overlapped characteristic peaks from the polymers are observed along with the characteristic peaks of MMT and GNPs in the region from 1700 to 500 cm^−1^ and this demonstrates the successful hydrogel formation through physical and chemical interactions.

Figure 2D–F shows the XRD patterns of pristine components and their hybrid hydrogels to evaluate their crystalline behavior before and after hydrogel formation. Figure 2D,E shows the XRD patterns of AM, PVA, SAG, MMT, and GNPs. In Figure 2D, AM exhibited some intense XRD peaks at 11.9°, 19.55°, 24°, 28.27°, 36.38°, and 49.21°. PVA showed major characteristic crystalline XRD peaks at 19.4°, 22.6°, and 40.3°, whereas SAG showed broad and weak XRD peaks at around 13.5° and 22°, demonstrating an amorphous structure [13]. For nanofillers (Figure 2E), MMT exhibited characteristic XRD peaks at 19.75°, 26.6°, 35.1°, and 42.4° [39], whereas GNPs showed an intense crystalline XRD peak at 26.3° and other characteristic XRD peaks were also observed at around 2θ = 43.2° and 54.8° [40]. The S1 hydrogel composed of PAM, PVA, and SAG exhibited a broad and amorphous peak, as compared to the pristine components, at around 20° (Figure 2F). It was speculated that microstructural changes occurred by breaking intra-molecular hydrogen bonds and making new inter-molecular hydrogen bonds, including entangled polymer chains. Furthermore, in other hydrogels, the crosslinking (S2) as well as the loading of MMT (S3) or MMT/GNPs (S4) did not show much any significant effect on the crystalline structure of the S1 hybrid hydrogel network, but exhibited some overlapped characteristic peaks (weak intensity).

### 2.3. Morphological Analysis

**Figure 3:**
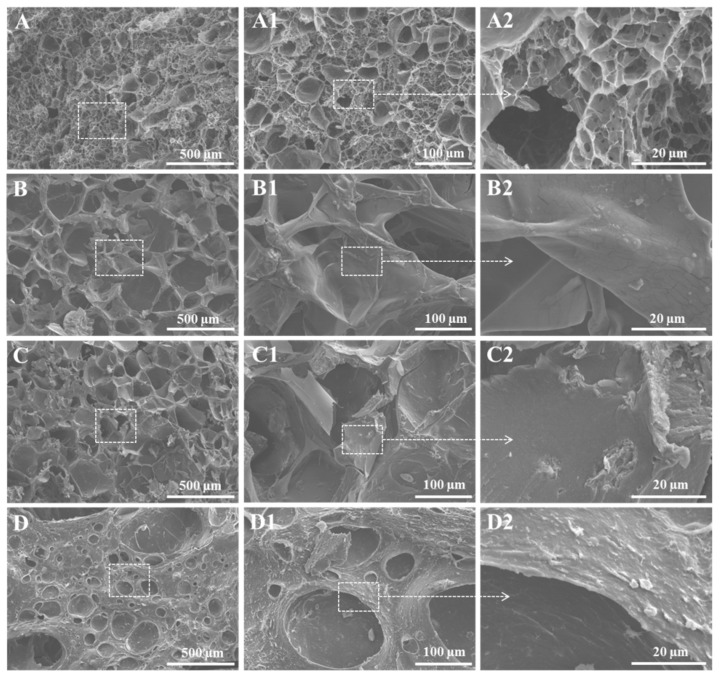
SEM image of crosslinked and nanofiller-reinforced hybrid hydrogels: S1 (**A**–**A2**), S2 (**B**–**B2**), S3 (**C**–**C2**), and S4 (**D**–**D2**), respectively.

Figure 3 shows the 3D morphology of the crosslinked hybrid hydrogels after removal of water molecules. Hydrogel S1 (A–A2) showed a decent porous surface morphology and good pore interconnectivity without using chemical crosslinking, but after imparting chemical crosslinking (both borax and ionic (Ca^2+^ ions)) in the hydrogel S2 (B–B2), an increased volume of the voids with thick pore walls was observed due to the synergistic effect of ionic (egg-box model) and borax crosslinking effects (reorganization of molecular chains). The effect of crosslinking the hybrid hydrogel S1 separately with borax and Ca^2+^ on the morphology is shown in Figure S2. Further, the loading of MMT into the hydrogel matrix (S3) exhibited less porous morphology with thick walls and dense microstructure compared to hydrogels S1 and S2, and it might be due to increased physical and chemical interactions among all components. Furthermore, by loading both MMT and GNPs into the hydrogel matrix (S4), it showed a denser microstructure and morphology with a low pore size with thick walls and low pore interconnectivity compared to all other hydrogels. Furthermore, the rough surface of hydrogels with surface-exposed MMT (C2) and GNPs (D2) particles could clearly be observed, which might have a positive role on cellular adhesion and viability.

### 2.4. Swelling and Degradation Behaviors

**Figure 4:**
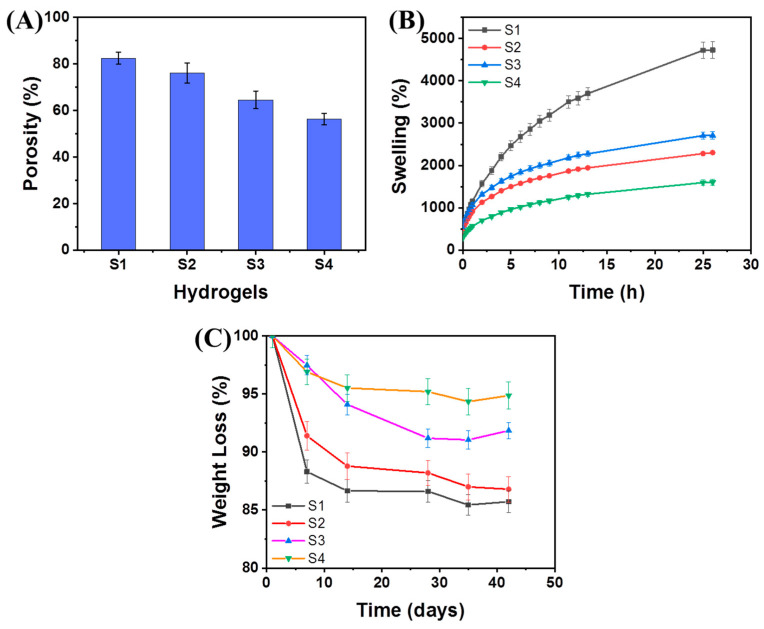
(**A**) Porosity (%); (**B**) swelling behavior (water) vs. time (h); and (**C**) degradation behavior (PBS) vs. time (days) for hybrid hydrogels.

Swelling and degradation behaviors are markedly important aspects of any hydrogel for appropriate cellular performance from adhesion to proliferation and differentiation for tissue regeneration. These aforementioned characteristics are dependent on various factors of the hydrogel network and forces, including the mesh size of polymer chains and the porosity (Figure 4A) [13]. The S1 hydrogel exhibited good porosity (~82.5%) and the porosity (%) of this hydrogel system was decreased with the incorporation of crosslinking (~76.1%) or the loading of MMT (~64.6%) or MMT/GNPs (~56.4%) in the hybrid hydrogel system, respectively. This reduction in porosity is due to enhanced physical and chemical crosslinking sites within the hydrogel network. Overall, the equilibrium swelling behavior of the hydrogel is dependent on three factors, including (1) polymer-water mixing; (2) osmotic pressure; and (3) internal elastic force acting on polymer chains [13,41]. All hydrogels exhibited good swelling behavior (i.e., water absorption capacity). As shown in Figure 4B, the S1 hydrogel showed higher swelling (%) and was almost saturated after 25 h of time period. The introduction of both borax and Ca^2+^ ion-crosslinking together in the hydrogel network (S2) decreased the swelling (%) greatly due to the synergic effect of both dynamic crosslinking mechanisms. However, the MMT clay-loaded hydrogel (S3) showed enhanced swelling (%), even rapid swelling behavior, and higher than that of the S2 hydrogel. Herein, we speculate that this might be due to the intrinsically hydrophilic nature of MMT and the loose crosslinking effect as compared to the unloaded hydrogel network. Further, the MMT/GNPs-loaded hydrogel (S4) exhibited a much reduced swelling (%) behavior than that of the S3 hydrogel. It could be due to the synergism of both nanomaterials as fillers through physical and chemical interactions and the surface hydrophobic nature of GNPs that prevented the high water absorption within the hydrogel network and delayed the time to attain equilibrium swelling. Furthermore, the swelling behavior can also be ascribed to complex pore-size formation (both in the bulk and at the surface) [13]. Overall, the S4 hybrid hydrogel demonstrated suitable water uptake capacity, which may be utilized promisingly in bone tissue engineering applications.

The 3D hybrid hydrogel shows a complex degradation pattern under a dynamic physiological environment and may differ fundamentally from one system to another [13]. Apart from a comprehensive in-depth study, our preliminary results of the degradation pattern of hybrid hydrogels in PBS are shown in Figure 4C. The degradation of the S1 hydrogel occurred due to two major pathways, (1) rapidly by solvation and (2) slowly by hydrolysis and diffusion of the hydrogel network. The crosslinking mechanisms (both borax and Ca^2+^ ions) slowed down the degradation pattern due to synergistic crosslinking moieties. Further, the loading of MMT or MMT/GNPs showed a reduced rate of degradation of hybrid hydrogels. Herein, it was speculated that the diffusion of PBS into these hydrogel networks is dependent on several factors, the degree and type of bonds, porosity, and the pore interconnectivity and their distribution in the hybrid hydrogels and in this course of action, the dissolution and hydrolytic reactions are undergone by the PBS.

### 2.5. Biomineralization Activity (In Vitro)

**Figure 5:**
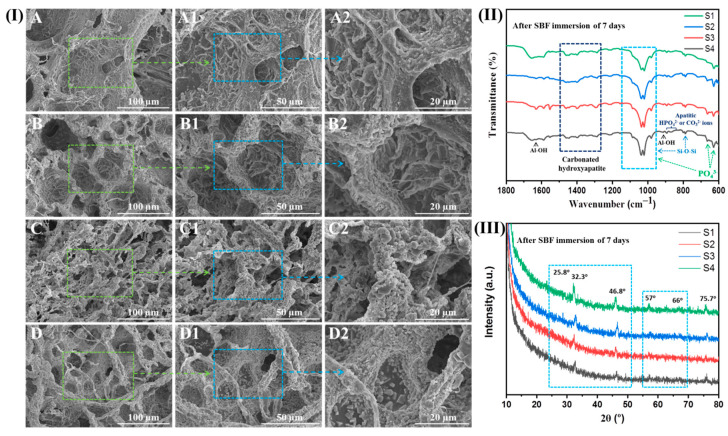
(**I**) SEM images of bone-like apatite formation on the surface of hybrid hydrogels after 7 days of SBF incubation: (**A**–**A2**) S1, (**B**–**B2**) S2, (**C**–**C2**) S3, and (**D**–**D2**) S4 hybrid hydrogels. (**II**) FTIR spectra and (**III**) XRD patterns of SBF-immersed hybrid hydrogels after 7 days of incubation.

All hybrid hydrogels were subjected to the simulated body fluid (SBF) solution for 7 days of incubation. The SEM micrographs are shown in Figure 5I to show the surface morphology of S1, S2, S3, and S4 hybrid hydrogels after SBF immersion post 7 days of incubation. After 7 days of SBF immersion, apatite precipitation was observed on the surface of the S1 hydrogel, including the deep pore surfaces. Furthermore, a high occurrence of apatite precipitates was found on the S2 hydrogel surface compared to the S1 hydrogels. However, a high apatite formation could be seen on the S3 hydrogel surface, wherein almost all of the hydrogel surface was fully covered by apatite (bone-like hydroxyapatite) which tended to be higher than S1 and S2 hydrogels. Furthermore, the S4 hydrogel also exhibited a large amount of apatite precipitates on its surface. The obtained results indicate the ability of S3 and S4 hydrogels to support apatite formation more than the native hydrogels, thereby supporting their capability to be used for bone tissue engineering.

In support of the bioactivity, hybrid hydrogels were also analyzed by FTIR for evaluating the apatite deposition on their surfaces. After 7 days of SBF immersion, FTIR spectra (Figure 5II) of hybrid hydrogels showed the characteristic phosphate (PO_4_^3−^) and carbonate (CO_3_^2−^) bands, hence supporting the confirmation of apatite layer deposition on hydrogel surfaces. Different modes of PO_4_^3−^ groups (absorption bands) are observed between 1100 cm^−1^ and 1000 cm^−1^ (broad peak due to the interaction of polymeric matrices and PO_4_^3−^ groups) and at 965 cm^−1^ (P-O stretching), and at around 670 cm^−1^ and 624 cm^−1^. Further, the characteristic absorption bands of CO_3_^2−^ groups are observed between 1475 cm^−1^ and 1295 cm^−1^, and around 874 cm^−1^ (that could emerge because of P-OH stretching from apatitic HPO_4_^2−^ ions or CO_3_^2−^ out-of-plane bending from apatitic CO_3_^2−^ ions) [13,42]. Furthermore, the characteristic peaks of polymeric matrices and nanofillers are overlapped and reduced due to the globular apatite deposition on hydrogel surfaces. Figure 5III shows the XRD patterns of S1, S2, S3, and S4 hybrid hydrogels where the broad amorphous peak for polymeric matrices was observed. After 7 days of exposure, the XRD patterns showed the characteristic peaks at around 2θ values of 25.8°, 27.9°, 29.4°, 31.8° (most intense), 32.9°, 46.8°, 49.4°, and 75.7° corresponding to the hydroxyapatite phase. In addition, two other major peaks were also observed at around 57° and 66° that are attributed to the amorphous calcium (i.e., CaCO_3_). Therefore, this confirmed the formation of carbonated hydroxyapatite through the likely substitution of CO_3_^2−^ groups by PO_4_^3−^ groups [43,44]. The XRD pattern of SBF-immersed hybrid hydrogels demonstrated the formation of apatite globular crystals (i.e., layer of calcium phosphate) similar to that of synthetic hydroxyapatite [13,45]. Moreover, after SBF immersion, the Ca^2+^, PO_4_^3−^ and MMT clay or MMT/GNPs within the hybrid hydrogel could effectively encourage the deposition Ca^2+^, PO_4_^3−^, and CO_3_^2−^ from SBF onto the surface of S1 and S2 hybrid hydrogels to form apatite globules of different sizes. Additionally, other ions from SBF could also be fused into the apatite globules (formed crystals). These elements altogether simulate the natural bone-like apatite in structure and composition that is beneficial for bone tissue regeneration. Moreover, the increased roughness and more porous surfaces of hybrid hydrogels after mineralization are very effective in increasing and speeding up cell attachment and tissue ingrowth [46].

### 2.6. Mechanical Properties

**Figure 6:**
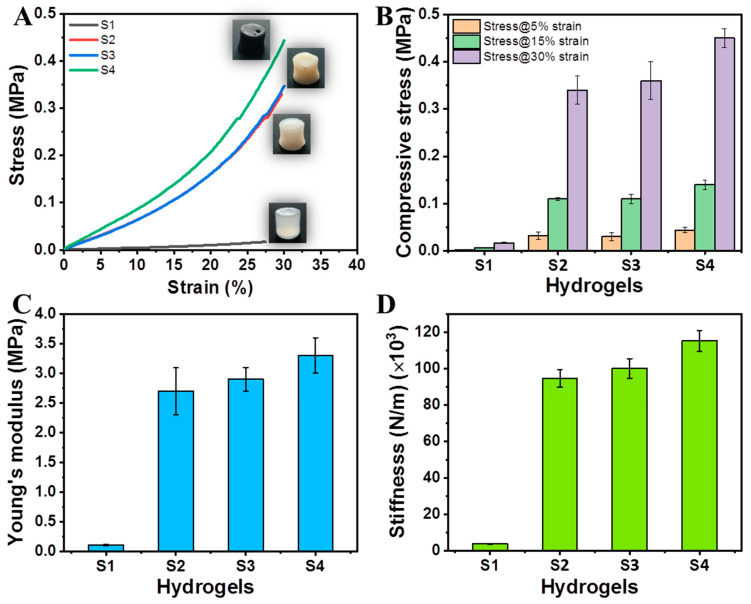
(**A**) Stress–strain curves; (**B**) compressive stress at three different strains (%); (**C**) Young’s modulus; and (**D**) stiffness values of hybrid hydrogels.

The impact of MMT or MMT/GNPs was evaluated on the compressive strength, Young’s modulus, and stiffness values of hybrid hydrogels. The compressive mechanical properties (i.e., strength, Young’s modulus, and stiffness) at maximum 30% of strain are shown in Figure 6A–D.

Hydrogel S1 showed a compressive strength of 0.017 ± 0.002 MPa and Young’s modulus of 0.11 MPa ± 0.01 without involving any of the crosslinking agents. However, by incorporating borax (B(OH)_4_^−^) and ionic (Ca^2+^) crosslinking together, hydrogel (S2) exhibited a remarkable improvement in compressive strength (0.34 MPa ± 0.03) and modulus (2.7 MPa ± 0.4). The effect of crosslinking the hybrid hydrogel S1 separately with borax and Ca^2+^ on the mechanical attributes is shown in Figure S3.

Further, the loading of MMT nanoclay into the hybrid hydrogel (S3) did not show much improvement in the compressive strength (0.36 MPa ± 0.04) and the modulus (2.9 MPa ± 0.2) compared to that of hydrogel S2. This might possibly be due to the issues of agglomeration and stress concentration sites due to the high amount of MMT. Furthermore, the loading of both MMT and GNPs as a binary system in the hybrid hydrogel (S4) enhanced the compressive strength (0.45 MPa ± 0.02) and the modulus (3.3 MPa ± 0.3), respectively. Herein, it is speculated that this binary exfoliated system (MMT/GNPs) facilitates decreased agglomeration and increased intercalation and exfoliation effects [47]. The stiffness values of S1, S2, S3, and S4 hydrogels are also shown in Figure 6D as 3761 ± 188, 94,518 ± 4725, 100,040 ± 5327, and 115,160 ± 5758 N/m, respectively. Moreover, the mechanical properties of the hybrid hydrogel can be modulated by using crosslinking agents and incorporating nanofillers.

### 2.7. Cytocompatibility

Initial cell–matrix interaction (adhesion), proliferation, and differentiation are important factors in tissue regeneration. Therefore, we investigated the in vitro cytocompatibility studies of as-produced hydrogels with the individual pre-osteoblast and fibroblast cell lines for a concluding aspect for utilized in-bone tissue engineering applications.

#### 2.7.1. Interaction of Hydrogels with Fibroblast Cells

**Figure 7:**
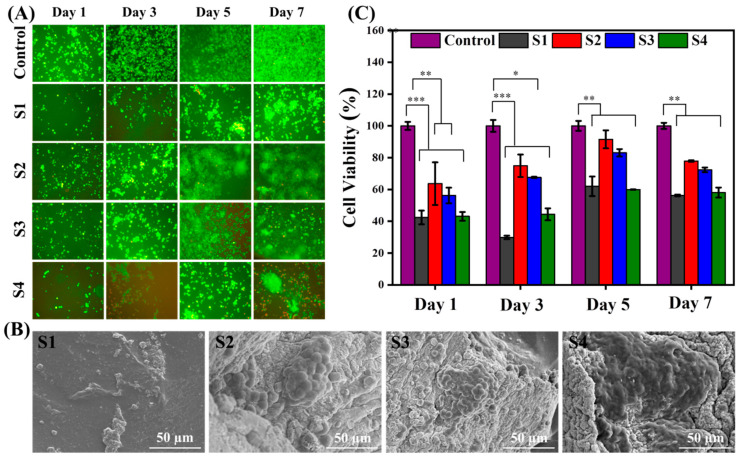
(**A**) Fluorescence images of live/dead assay; (**B**) SEM images of S1, S2, S3, and S4 hydrogels after 7 days of cell culture; and (**C**) MTT assay for control and S1, S2, S3, and S4 hydrogels, respectively, after 1, 3, 5, and 7 days of cell culture (L929 fibroblast). *** *p* ≤ 0.001, ** *p* ≤ 0.01, * *p* ≤ 0.05.

The interaction of fibroblast cells with the developed hybrid hydrogels was evaluated using qualitative (live/dead assay and SEM images) and quantitative (MTT assay) analyses. For day 1, in live/dead assay (Figure 7A), the S1 hydrogel exhibited some cytotoxicity because fibroblast cells are exposed to the 3D hydrogel network as well as residual cytotoxic elements (i.e., impurities) if any, instead of the control. This is apparently due to a new 3D hydrogel environment with more space and a higher surface area, wherein cells take time to accommodate this new 3D environment for adhesion, migration, and proliferation. Therefore, an extended proliferation activity or survival ability of cells for a long- duration was observed compared to the control. When compared to the crosslinked hydrogel (S2), the cell viability and proliferation was enhanced and this might be due to the increased stiffness of the hydrogel network. However, the loading of MMT into the hydrogel system (S3) exhibited decreased cell viability due to the complex nature of mechanical stiffness and clay particles. Similarly, the loading of a binary system of MMT/GNPs into hydrogel system (S4) showed further decreased cell viability compared to the S3 hydrogel. In addition, SEM images are also shown for morphological cell adhesion on hybrid hydrogels after 7 days of culture (Figure 7B). A similar trend in the cell behavior was also observed for MTT assay (for 1, 3, 5, and 7 days of cell culture; Figure 7C). The effect of crosslinking the hybrid hydrogel S1 separately with borax and Ca^2+^ on the cellular behavior is shown in Figure S4.

#### 2.7.2. Interaction of Hydrogels with Pre-Osteoblast Cells

**Figure 8:**
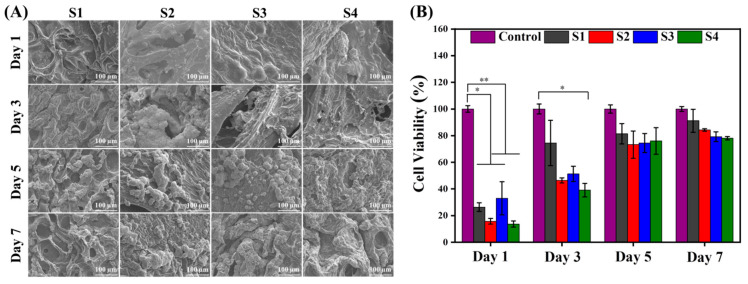
(**A**) SEM images and (**B**) MTT assay of S1, S2, S3, and S4 hydrogels after 1, 3, 5, and 7 days of cell culture (pre-osteoblast cells). ** *p* ≤ 0.01, * *p* ≤ 0.05.

Four diverse hydrogels were cultured with pre-osteoblast cells for 1, 3, 5, and 7 days for comparative cellular responses and evaluated. Based on qualitative analysis (SEM images in Figure 8A), it can be observed that S1 hydrogels showed sufficient cell adhesion on the hydrogel surface for 1 day of cell culture and the spreading over the whole surface was increased for further 3, 5, and 7 days, respectively. Chemically crosslinked hydrogel (S2) using B(OH)_4_^−^ (aq) and Ca^2+^ ions exhibited an accelerated cellular adhesion and proliferation from 1 to 7 days of cell culture. In addition, the MMT-loaded hydrogel (S3) showed more frequent cellular adhesion and proliferation as compared to the S2 hydrogel. Furthermore, the MMT/GNPs-loaded hydrogel (S4) showed little cytotoxicity and thereby cellular activity. Herein, it is assumed that GNPs could cause some cytotoxicity to pre-osteoblast cells due to the impurities that originated during the synthesis process.

To further evaluate this cellular behavior of the hydrogels in a quantitative prospective, an MTT assay was performed (Figure 8B). The MTT assay exhibited good metabolic activity of pre-osteoblast cells in the S1 hydrogel, but lower than that of the control, which is possibly due to the 3D exposure of cells with hydrogel and some residual cytotoxic impurities (if any). Upon introduction of the crosslinking functionalities, some cytotoxic effect was observed in the S2 hydrogel as compared to the S1 hydrogel which could be due to the residual Ca^2+^ ions in the hydrogel network. However, the incorporation of MTT in the hydrogel (S3) initially enhanced the cellular metabolic activity, which was further reduced to some extent after 3 days and followed a similar decreasing trend to 5 and 7 days of culture. Furthermore, MMT/GNPs-loaded hydrogel (S4) showed a lower cytocompatibility initially as compared to the S3, S2, and S1 hydrogels and followed the successive trend for days 3, 5, and 7, respectively. We speculate that this effect might be related to the size- and dose-dependent cytotoxicity of GNPs and the residual Ca^2+^ ions in the hydrogel network.

## 3. Materials and Methods

### 3.1. Materials

Poly (vinyl alcohol) (PVA, degree of polymerization: 1500, average molecular weight: 66,000) and borax (BX) were purchased from Duksan Pure Chemical Co., Ltd. (Seoul, Republic of Korea), sodium alginate (SAG) was supplied by Wako Pure Chemical Industries Ltd. (Osaka, Japan), graphene nanoplatelets (GNPs) were procured from Asbury Mills Inc. (Asbury, NJ, USA), and montmorillonite K10 (MMT K10, surface area 220–270 m^2^/g) clay, acrylamide (AM, 98%), N,N′-methylene-bisacrylamide (MBA, 99% purity), sodium chloride (NaCl), potassium chloride (KCl), calcium chloride (CaCl_2_·2H_2_O), sodium bicarbonate (NaHCO_3_), disodium phosphate (Na_2_HPO_4_·2H_2_O), magnesium chloride (MgCl_2_·2H_2_O), sodium sulfate (Na_2_SO_4_), Tris buffer (CH_3_OH_3_-CNH_2_), hydrochloric acid (HCl), and 3-(4,5-dimethylthiazol-2-yl)-2,5-diphenyl-2H tetrazolium bromide (MTT) were procured from Sigma-Aldrich Co. (Missouri, United states of America). Ammonium persulfate (APS) was procured from Dae-Jung Chemical and Metal Co., Ltd. (Gyeonggi-do, Republic of Korea). Human skin fibroblast (CCD-986Sk) and pre-osteoblast (MC3T3-E1) cell lines were purchased from the Korean Cell Bank. All reagents and chemicals were of analytical grades and used as received. All the experimental procedures were performed using deionized water (DW).

### 3.2. Preparation of Hybrid Hydrogels

For synthesizing PAM/PVA-SAG (S1) hybrid hydrogel, PVA (2 g) and SAG (2 g) were homogenously dissolved in 70 mL DW, whereas AM (9.8 g) was dissolved in 25 mL DW, and then mixed together to form a homogenous hybrid solution. Afterwards, the desired volumes (2.5 mL) of MBA (0.2 g) and APS (0.1 g) solutions were added to the prepared hybrid solutions consecutively and amalgamated completely. Then, this solution was poured into 24-well culture plates (mold) and 3 freeze–thawing cycles under room temperature and −20 °C to improve physical interactions (hydrogel bond) were completed. Finally, the hybrid solution was kept in an oven maintained at 60 °C for 5 h to initiate the free-radical polymerization process. After polymerization, samples were taken out and washed with DW to remove unreacted monomer or chemical species. Further, S1 hydrogel was crosslinked by immersing in the mixed solution of borax and CaCl_2_ and obtained PAM/PVA-SAG/BX-Ca^2+^ (S2) hybrid hydrogel. Similarly, PAM/PVA-SAG/MMT/BX-Ca^2+^ (S3) hybrid hydrogel was prepared by dispersing MMT clay (10 wt.%, 1.4 g) into DW (25 mL) and sonicated for 30 min to make a homogenous dispersion solution and dissolved AM (9.8 g) into this MMT-dispersed solution. Then, the same procedure was followed, as described above. However, after mixing AM/MMT dispersion solution and PVA/SAG solution (70 mL), the composite solution was again sonicated for 30 min for homogenous dispersion of MMT clay. Further, PAM/PVA-SAG/MMT-GNPs/BX-Ca^2+^ (S4) hybrid hydrogel was similarly prepared, except the dispersion of MMT (10 wt.%) and GNPs (1 wt.%, 0.14 g) together in DW. The digital images of the obtained hybrid hydrogels and their proposed reaction mechanisms are shown in Figure 1. Finally, these samples were kept in refrigerator (−20 °C) until further use. The synthesis process of different hybrid hydrogels is summarized in Table 1.

### 3.3. Characterization

#### 3.3.1. Morphology

Morphology of the hydrogels was analyzed with the help of scanning electron microscopy (SEM; HITACHI S4800, Tokyo, Japan). Before analysis, hydrogels were equilibrated with distilled water and freeze dried (−80 °C). Samples were mounted on the stub and then coated with a thin layer of platinum at low deposition rate.

#### 3.3.2. Porosity

Porosity (%) of the hydrogels was calculated by the liquid immersion process [48]. In brief, hybrid hydrogels were freeze-dried and then the remaining moisture was removed by vacuum drying. Then, these dried hydrogels were immersed into absolute ethanol until saturation, removed, and the excess ethanol was wiped out by using tissue paper, weighed, and then the porosity was measured by the procedure as given elsewhere [48].

#### 3.3.3. Fourier Transform Infrared Spectroscopy (FTIR)

Functional groups of the components and their interactions in hybrid hydrogels were evaluated by using Fourier-transform infrared spectroscopy (FTIR; Perkin Elmer) under transmittance mode between 4000–500 cm^−1^.

#### 3.3.4. X-ray Diffraction (XRD)

The crystalline behavior of pristine components and their corresponding hydrogels was measured by X-ray diffraction (XRD, Bruker AXS d8 Advance) with λ = 1.54 Å (CuKα radiation at 40 kV and 30 mA). Here, the XRD patterns were measured at 2θ = 10–70° (angular range) with a 10°/min scanning rate.

#### 3.3.5. Relative Swelling and Degradation Behaviors

Relative swelling behavior (%) was evaluated by submerging hydrogels (dried) in phosphate-buffered saline (PBS) until saturation. Further, fully swollen hydrogels were removed and excess PBS was wiped out using a tissue paper and then weighed. Relative swelling ratio (%) was determined by using the formula:

(1) $$\mathrm{Relative}\;\mathrm{swelling}\;\mathrm{ratio}\;\left(\%\right)=\frac{W_{s}-W_{d}}{W_{d}}\times 100$$

where *W_s_* and *W_d_* are the weights of swollen and dried hydrogels, respectively. Three independent experiments were carried out for each sample [48].

For degradation analysis, hydrogels were submerged in PBS (pH, 7.4) to determine in vitro degradation by measuring weight loss at 37 °C. For pre-determined time periods, samples were incubated and taken out, washed, freeze-dried, and then weighed. The degradation (%) was measured using the following formula:

(2) $$\mathrm{Degradation}=\frac{W_{o}-W_{d}}{W_{o}}\times 100$$

where *W_o_* = original weight; and *W_d_* = freeze-dried weight of the hydrogels after immersing in PBS for pre-determined time periods [48].

#### 3.3.6. Mechanical Testing

Compressive mechanical properties were analyzed by using a universal testing machine (UTM; Lloyd Instruments, West Sussex, UK) for cylindrical samples (diameter 20 mm and height 9 mm) up to 30% strain (deformation) at a strain rate of 2 mm/min.

#### 3.3.7. In Vitro Bioactivity

The biomineralization activity in vitro (i.e., apatite-forming ability) was performed by submerging hydrogels in simulated body fluid (SBF) solution for a time period of 7 days under static conditions. Prior to analysis, Tris-HCl-buffered SBF solution (27 mM HCO_3_^−^) was prepared comprising of an ionic composition [49,50] similar to human blood plasma. Further, ~200 mg of hydrogels was immersed in bottles containing 50 mL SBF solution and kept at 37 °C for pre-determined time periods and SBF was changed for alternative days. After desired time periods, hydrogels were removed and washed with ethanol/deionized water 2–3 times followed by freeze drying (−80 °C) for 24 h. The hydroxyapatite-like apatite formation on hydrogel surfaces was analyzed by SEM/EDX, FTIR, and XRD analyses for morphological and structural analyses.

#### 3.3.8. In Vitro Cytocompatibility

Hydrogels were seeded with pre-osteoblast and fibroblast cells separately for SEM analysis (cell adhesion and spreading), Live/Dead assay (cell viability), and MTT assay (cell proliferation).

##### Cell Adhesion and Spreading

For cell culture, osteoblast cells (MC3T3-E1) were harvested in Minimum Essential Media (MEM α), 10% Fetal Bovine Serum (FBS), and 1% antibiotic combination and maintained in a humidified incubator (37 °C, 5% CO_2_). Before seeding of cells, hydrogels were sterilized by saturating in ethanol (70%) followed by disinfection under UV for 30 min and washing with PBS (three times). Then, selected hydrogels were placed in each pre-determined well of the 24-well plates (two sets) and growth media was added followed by keeping them in a humidified incubator for 4 h. The cell media was taken out and fibroblast and osteoblast cells (5 × 10^4^ cell/well) were carefully seeded on hydrogels, in two separate plates. Then, the 24-well plate was placed into a humidified incubator for bone-cell growth. For morphological analysis, both fibroblast (CCD-986Sk) and osteoblast cells (MC3T3-E1) (5 × 10^4^ cells/well) were seeded on hydrogels in 24-well plates (separately) and then incubated in humidifier incubator for 1, 3, 5, and 7 days. The media was removed and hydrogels were washed with PBS, and then cells were fixed by using 4% paraformaldehyde solution for 15 min. Finally, hydrogels were washed with PBS, dehydrated with ethanol–water mixtures, and then freeze-dried and characterized by using SEM.

##### Cell Viability

Quantitative viability of fibroblast cells (CCD-986Sk) and pre-osteoblast cells (MC3T3-E1) on hydrogels was measured by using cell proliferation assay MTT. For proliferation assay, cell-seeded hydrogels were incubated for 1, 3, 5, and 7 days in a humidified incubator. After desired time periods, 200 μL MTT (5 mg/mL in 7.4 PBS) was added to each pre-determined well and again incubated for 4 h in a humidified incubator. Next, media was removed and formazan crystals were solubilized in isopropanol (acidified) and subsequently analyzed using microplate reader (Bio-T Instruments) at 570 nm wavelength. In addition, the qualitative viability of MC3T3-E1 cells and CCD-986Sk cells on hydrogels was evaluated by using Live/Dead assay. In brief, cells (5 × 10^4^ cells/well) were seeded on the hydrogels and incubated in the incubator humidifier for 3, 7, and 14 days. After the specified time periods, the media was replaced with 200 μL of ethidium homodimer-1/calcein-AM (20 μL of ethidium homodimer-1 and 2 μL of calcein-AM in 10 mL PBS) and incubated for 30 min in dark at room temperature. Finally, these pre-osteoblast cells fluorescently were visualized in hydrogels by fluorescence microscopy (Nikon Eclipse 8 Ti).

#### 3.3.9. Statistical Analysis

Three independent experiments were conducted and data are expressed as mean ± standard deviation (SD), indicating error bars. Statistical analysis was completed using Graph pad, Prism (version 5.1) software. The data were statistically analyzed using two-way analysis of variance (ANOVA) at *p* < 0.01 level of significance.

## 4. Conclusions

Hydrogels are effective in providing a 3D environment for regenerating damaged tissue. However, due to low mechanical performance and insufficient biomineralization behavior of the hydrogels, the manufacturing of functional hybrid hydrogels is imperative to facilitate cellular response, and thereby the satisfactory tissue regeneration. In this study, we prepared crosslinked nanocomposite hydrogels composed of PAM, PVA, and SAG with MMT or MMT/GNPs as nanofillers through chemical (BX and Ca^2+^ ions) and physical crosslinking. Hydrogels showed good interconnected porous microstructures and good compressive mechanical properties. A significant improvement in compressive mechanical strength (0.36 MPa ± 0.04 MPa or 0.45 MPa ± 0.02 MPa) and Young’s modulus (2.9 MPa ± 0.2 MPa or 3.3 MPa ± 0.3 MPa) of the nanocomposite hydrogels was observed when reinforced with MMT (S3) or MMT/GNPs (S4), respectively. Enhanced bioactivity (i.e., biomineralization) was measured for MMT-loaded (S3) and MMT/GNPs-loaded (S4) hydrogels. Moreover, a satisfactory cytocompatibility behavior was observed for all hydrogels as investigated from day 1 to day 7 of cell culture with fibroblast and pre-osteoblast cell lines. Overall, MMT-loaded (S3) or MMT/GNPs-loaded (S4) hydrogels exhibited good cellular behavior. These results revealed that as-developed hybrid hydrogels could further be explored for comprehensive in vivo biological analyses for suitable tissue regeneration.

## Figures and Tables

**Table 1:** Composition of the hydrogels.

Composition	AM (g)	PVA (g)	SAG (g)	MBA (g)	APS (g)	MMT (g)	GNPs (g)	BX/Ca^2+^
S1	9.8	2	2	0.2	0.1			-
S2	9.8	2	2	0.2	0.1			+
S3	9.8	2	2	0.2	0.1	1.4		+
S4	9.8	2	2	0.2	0.1	1.4	0.14	+

## Data Availability

Not applicable.

## Supplementary Materials

The following supporting information can be downloaded at: https://www.mdpi.com/article/10.3390/ijms232214158/s1.
