# Supplementary material for: Triple-Networked Hybrid Hydrogels Reinforced with Montmorillonite Clay and Graphene Nanoplatelets for Soft and Hard Tissue Regeneration
Source: Int J Mol Sci. 2022 Nov 16;23(22):14158. doi: 10.3390/ijms232214158 (PMC9698198; doi:10.3390/ijms232214158)
Supplement: Supplementary file 1 [file ijms-23-14158-s001.zip › ijms-1993366-supplementary.pdf]

Structural analysis

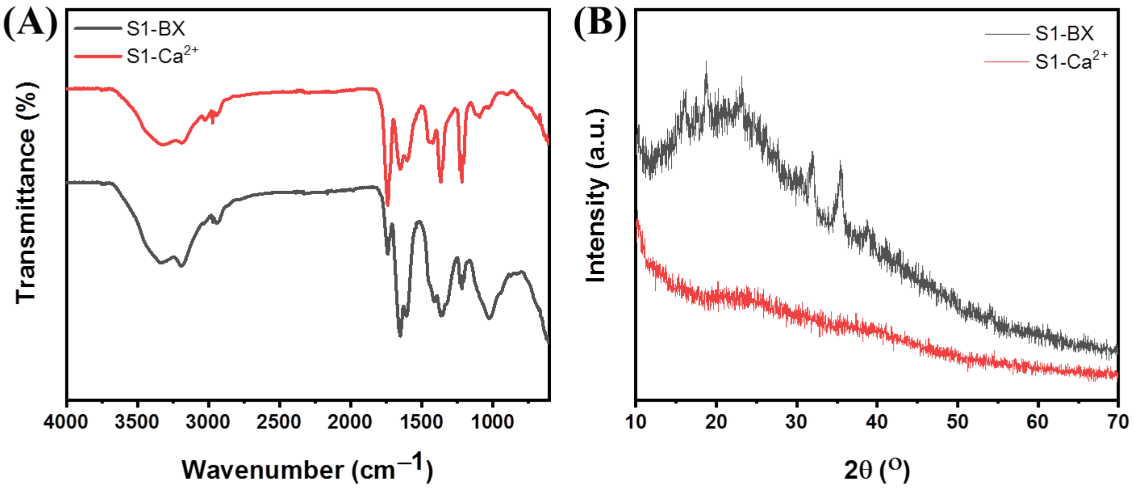

Figure S1. (A) FTIR spectra and (B) XRD pattern of borax-crosslinked and Ca^{2+} ions-crosslinked S1 hybrid hydrogels.

Morphology

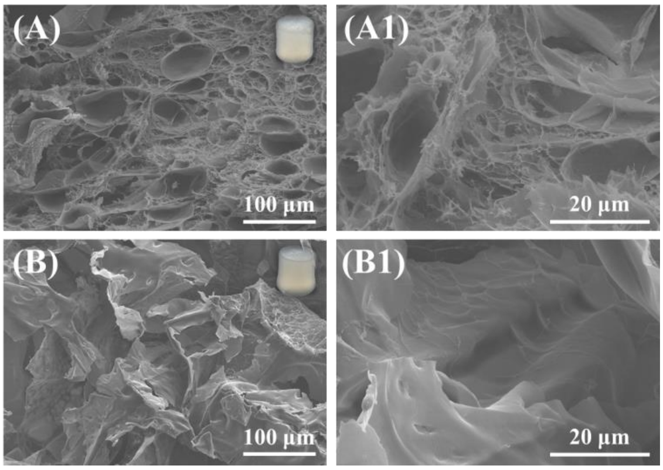

Figure S2. SEM images of (A-A1) borax-crosslinked and (B-B1) Ca^{2+} ions-crosslinked S1 hybrid hydrogels.

Mechanical analysis

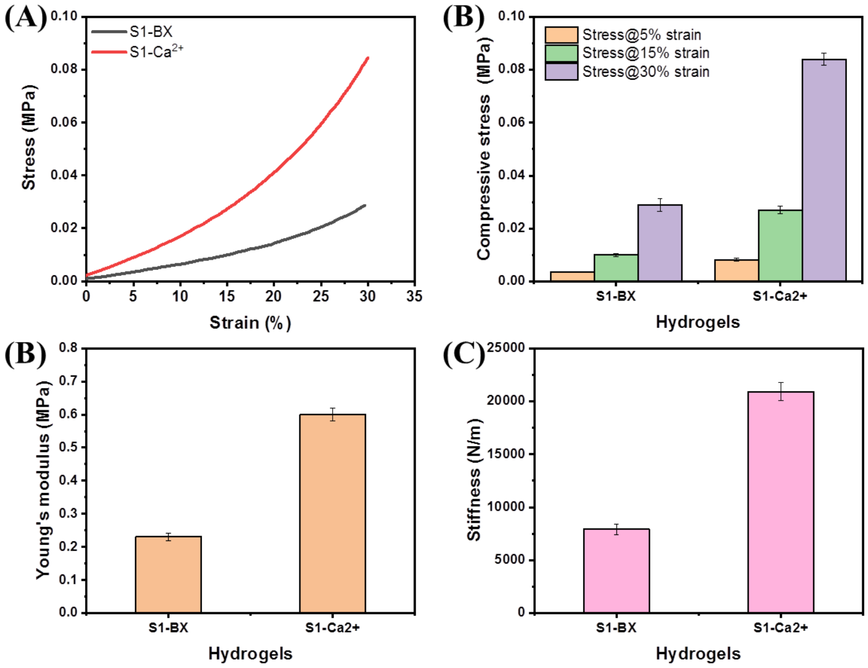

Figure S3. (A) Stress-strain curves, (B) compressive strength, (C) Young's modulus, and (D) stiffness values of borax-crosslinked and Ca^{2+} ions-crosslinked S1 hybrid hydrogels.

In-vitro Cytocompatibility

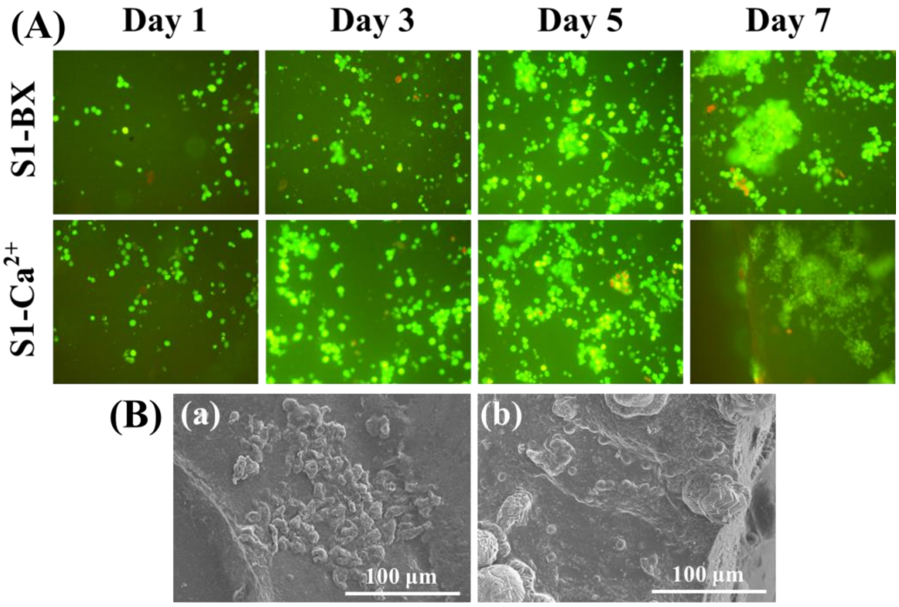

Figure S4. (A) Fluorescence images of live/dead assay of borax-crosslinked and Ca^{2+} ions-crosslinked S1 hybrid hydrogels after 1, 3, 5, and 7 days of culture with fibroblast cells. (B) SEM images of borax-crosslinked and Ca^{2+} ions-crosslinked S1 hybrid hydrogels after 7 days of culture with fibroblast cells.
